# *Coxiella burnetii* Infection in Cats

**DOI:** 10.3390/pathogens12121415

**Published:** 2023-12-02

**Authors:** Valentina Virginia Ebani

**Affiliations:** 1 Department of Veterinary Sciences, University of Pisa, Viale delle Piagge 2, 56124 Pisa, Italy; valentina.virginia.ebani@unipi.it; 2 Centre for Climate Change Impact, University of Pisa, Via del Borghetto 80, 56124 Pisa, Italy

**Keywords:** *Coxiella burnetii*, Q fever, cat, zoonosis, epidemiology

## Abstract

Q fever is a zoonotic disease caused by *Coxiella burnetii*, with farm ruminants being considered the main sources of infection for humans. However, there have been several cases of the disease in people that have been related to domestic cats as well. Cats can become infected through various routes, including ingestion of raw milk, hunting and consuming infected rodents and birds, consumption of contaminated pet food, inhalation of contaminated aerosols and dust, and bites from hematophagous arthropods. Infected cats typically do not show symptoms, but pregnant queens may experience abortion or give birth to weak kittens. Accurate diagnosis using serological and molecular methods is crucial in detecting infected cats, allowing for prompt action with appropriate treatments and preventive measures. Breeders, cattery personnel, veterinarians, and owners should be informed about the risks of *C. burnetii* infections associated with cats experiencing reproductive disorders.

## 1. Introduction

*Coxiella burnetii* is the etiologic agent of the zoonosis called Q fever. The disease was first described in Australia in 1935 by Edward Holbrook Derrick who investigated a disease in a group of abattoir workers in Brisbane, Queensland, Australia. The “Q” comes from “query” fever, as named by Derrick [1].

Q fever affects various domestic and wild mammals, which act as reservoirs for the infection and pose a severe public health threat. The pathogen was detected in birds, reptiles, and arthropods as well. Farm ruminants are considered the main reservoirs which often act as the major contributors to the transmission of the pathogen to humans [2]. The main transmission routes, for humans and animals, of C. burnetii are the inhalation of aerosols or dust containing the microorganisms, and the ingestion of contaminated food [2]. 

Cats are known to be susceptible to *C. burnetii* as well, even though their role in the epidemiology of this infection has not been fully elucidated [3].

Cats are popular pets globally, and in many countries, there are more domesticated felines than domesticated dogs [4]. The risk of transmission of zoonotic pathogens from cats to humans is high. Cats are responsible for a large number of animal bites, which often become infected. Zoonotic pathogens can also be transmitted through contact with infected cats’ saliva or other excretions, contaminated vehicles such as food, water, and fomites, as well as shared vectors or environmental exposures [5]. Even though domestic cats can be potential sources of numerous infectious disease agents, many of these diseases can be controlled through routine veterinary care, proper vaccination regimens, and parasite treatment. However, free-roaming cats often lack the necessary preventive care to control these diseases, posing a potential health threat to other domestic animals, wildlife, and humans [6].

The aim of this narrative review is to draw attention to the key aspects of *C. burnetii* infection in cats, with a particular emphasis on the role of these animals in the epidemiological cycle of Q fever.

## 2. Etiology

*Coxiella burnetii* is a Gram-negative bacterium belonging to the family Coxiellaceae, order Legionellales. It is a pleomorphic rod, of small dimensions (0.2–0.4 μm wide, 0.4–1.0 μm long), occurring as two different forms: a large-cell variant (LCV) and a small-cell variant (SCV). The LCV is the larger, less electron-dense and metabolically active intracellular form of the pathogen; a sporogenic differentiation of the LCV produces the SCV that is a resistant spore-like form. SCV is released when the cells lyse and can survive for long periods in the environment [7]. 

SCV can survive for several weeks to months lying idle in the soil, capable of surviving standard disinfectants and resisting heating or drying. For example, it can survive 7–10 days on wool at room temperature, 1 month on fresh meat in cold storage, 120 days in dust, and more than 40 months in skimmed milk [8]. Moreover, it resists elevated temperatures, desiccation, osmotic shock, ultraviolet light, and chemical disinfectant [9].

*Coxiella burnetii* lives and multiplies in monocytes, macrophages, and trophoblasts of the host [10]; as the microorganism multiplies, it destroys the host cell and moves on to live in other cells [8].

*Coxiella burnetii* has antigenic variations related to mutational variations in the lipopolysaccharide (LPS). Phase I, corresponding to smooth LPS, is highly infectious and naturally present in infected animals. Phase II, showing a truncated LPS and without some protein cell surface determinants, corresponds to rough LPS; it is not very infectious and is obtained only in laboratories after serial passages in embryonated egg or cell cultures [11,12]. Antibodies to phase I antigens of *C. burnetii* generally require longer to appear and indicate continued exposure to the bacteria. Therefore, increased antibodies in a serum sample to phase II antigens indicate acute cases, while a rise in phase I reflects a chronic infection of Q fever [8].

## 3. Epidemiology

*Coxiella burnetii* can infect various domestic and wild animal species, including mammals, birds, and reptiles. However, the primary reservoirs of the pathogen are considered to be cattle, sheep, and goats. In these animals, coxiellae primarily cause reproductive disorders, leading to significant economic losses. Infected animals shed coxiellae through aborted fetuses, placentas, lochiations, urine, feces, and milk [13].

It is also known that dogs and cats are susceptible to *C. burnetii* infection. Pet animals, especially those in close contact with their owners, have been suspected of acting as reservoirs of *C. burnetii* during urban Q fever outbreaks [13]. 

### 3.1. Q Fever Human Cases Related to Infected Cats

Q fever cases in humans associated with direct or indirect contact with *C. burnetii*-infected cats have been reported since the 1980s.

Kosatsky, in 1984 [14], well described a case of Q fever in a family living in Nova Scotia (Canada). The family members and their friends developed a febrile respiratory disease also characterized by bradycardia and palatal petechiae. Complement fixation tests detected antibodies against *C. burnetii* phase II antigen in the serum of the patients. Investigations showed that the patients had entered the home where the family cat, subsequently found to have antibody to the pathogen, had given birth to kittens and nursed them in a basket kept inside the entryway [14]. 

Some years later, Marrie et al. [15,16] suspected a strong association between human Q fever and exposure to stillborn kittens and parturient cats, on the basis that numerous cases of infection occurred in people living in Nova Scotia. An interesting case of human Q fever outbreak was observed in 1985 in Baddeck, a village in northeastern Nova Scotia. Fourteen residents lived or worked in four buildings located side by side in the center of the village. Most of them were exposed to a cat that gave birth to stillborn kittens and had bloody vaginal discharge for three weeks prior to delivery. The female cat, which resulted serologically positive to *C. burnetii* phase I and II antigens, lived in one of the four buildings, but frequently visited the other three ones [16].

A Q fever outbreak occurred in Halifax, Nova Scotia, when a group of poker players developed pneumonia within a few days; Q fever was diagnosed and epidemiological investigations discovered that the infection originated from a female cat who lived in the house where the players met regularly [17]. 

Marrie and collaborators, in 1989 [18], described a Q fever case affecting 16 of 32 employees at a truck repair plant in Truro, Nova Scotia. None of the affected men have had direct or indirect contact with cattle, sheep, or goats, but one of the workers had a cat who gave birth to kittens two weeks prior to the first case of Q fever. The cat owner fed the kittens every day before coming to work as the cat would not let the kittens suckle. Serological diagnosis on cat’s serum found antibodies to *C. burnetii* phases I and II antigens. Q fever was also developed by the cat owner’s wife and son, whereas none of the family members of the other employees with Q fever were affected. It is interesting to note that among the sixteen infected men, only one had direct contact with the queen cat; therefore, it seems plausible that the infection in the other employees was due to exposure to contaminated clothing of the cat owner [18]. After all, clothing contaminated by *C. burnetii* bacteria has been considered a source of infection in previously observed human cases of Q fever [19,20,21].

The relation between *C. burnetii*-infected cats and human cases of Q fever were also demonstrated by outbreaks in other countries. In Eastern Maine (USA), members of a family were exposed to a parturient cat during a reunion; after two weeks, they developed clinical signs referable to Q fever. The serological diagnosis confirmed *C. burnetii* infection in the affected people and the family cat [22]. 

A Q fever outbreak occurred among the staff members of a small animal veterinary hospital in Sidney (Australia). Nine veterinary personnel were confirmed to have cases of Q fever on the basis of positive results obtained by serological and/or PCR tests. Of the nine veterinary personnel, eight had worked on the day a caesarean section was performed on a queen, while the ninth person handled the equipment used during the caesarean section the following morning [23].

Similarly, Malo and collaborators, in 2018 [24], described a human outbreak of Q fever in southeast Queensland (Australia): two individuals working in a veterinary clinic and four workers of an animal refuge developed diseases after exposure to a parturient queen cat and her litter that were euthanized the same day as the birthing event. Laboratory diagnosis, through serological and molecular methods, confirmed *C. burnetii* infection in the patients.

### 3.2. Epidemiological Surveys in Cats

The role of cats in the epidemiology of Q fever has been studied through serological investigations for several years; successively, when molecular methods became available, studies have been carried out, also searching for *C. burnetii* DNA in feline samples. 

In all case, the prevalence values found in the different surveys were difficulty comparable, because they were related to several factors such as geographical area, feline population, environmental conditions, tests, and antigens employed in the diagnosis.

In 1970s, Randhawa and coworkers [25] found that 19.8% of pound cats from southern California (USA) had antibodies to *C. burnetii* phase I antigen, testing the animal sera with the capillary agglutination test. Willeberg et al. [26] detected 9% of stray cats, from a different area of California, with antibodies to *C. burnetii* phase II antigen using the microagglutination test.

More recently, Cairns et al. [27] carried out a study to determine the prevalence of *C. burnetii* DNA in uterine and vaginal tissues from healthy, client-owned and shelter cats of north-central Colorado (USA) using PCR; a 0% prevalence was found in shelter cats, while 8.5% (4/47) pet cats resulted infected.

In Nova Scotia, 216 cats were tested for *C. burnetii* infection by indirect immunofluorescence assay; 24.1% of the animals had antibodies to phase II antigen and 6% to phase I antigen. Interestingly, none of the 447 dogs from the same geographic area tested during the same investigation had antibodies to the pathogen [28]. Successively, cats from two different provinces in Canada were analyzed, and seroprevalences of 7.2% (6/97) and 19.4% (20/104) were observed, confirming the circulation of *C. burnetii* in cat populations in this country [29].

In addition, a total of 184 cats were recently tested in Quebec (Canada): 59 from ruminant farms, 73 pets, and 52 feral cats. All pets and feral cats were negative to *C. burnetii* with ELISA and qPCR, while among farm cats, 2/59 (3.4%) were ELISA positive, 3/59 (5.1%) were ELISA doubtful, and 1/59 (1.7%) (rectal swab) was qPCR-positive. Farm cat positivity was associated with a positive *C. burnetii* status on the ruminant farm where the tested cats lived [30].

Data about *C. burnetii* exposure in cats in Asia come from a survey by Morita et al. [31] who found a 16% (16/100) seroprevalence among domestic cats in Japan. A more recent research showed the circulation of *C. burnetii* among cats in Japan and Korea, with different scenarios between stray cats and pets; seroprevalences of 0% and 8.6% (10/116) were detected in stray and pet cats, respectively, in Korea, whereas a higher seroprevalence was detected in stray cats (15/36; 41.7%) relative to pet cats (44/310; 14.2%) in Japan. The higher prevalence detected in stray cats suggested the consumption of wild birds and rodents, more frequent in stray animals, as a relevant risk of infection [32]. Similar results, which corroborated this hypothesis, were found in Iran where a seroprevalence of 22.35% (19/85) and 11.53% (9/78) were detected in stray and pet cats, respectively [33].

Moreover, a study was carried out in stray cats from three providences (Ankara, Niğde, and Kayseri) in Central Anatolia, Turkey. A total of 143 sera were examined for the presence of IgG against *C. burnetii* phase II antigen by an indirect fluorescent antibody test, and seven (4.9%) cats resulted positive, even though different seroprevalences were observed in relation to the provinces where the tested animals lived [34].

*Coxiella burnetii* seems to circulate in feline populations in other Asian areas, too. A low seroprevalence (0.51%) was recently found in cats from Thailand; indirect immunofluorescence tests detected antibodies to *C. burnetii* phase I and II antigens in 2 cats of the 390 tested, all residing in communities far from cattle farms [35].

The first information about the exposure of cats to *C. burnetii* in the African continent has been reported by Matthewman et al. [36], who detected 2% (1/52) of seropositive cats in South Africa and 13% (15/119) in Zimbabwe in a survey performed testing sera by indirect immunofluorescence with phase I antigen. More recently, Abdel-Moein and Zaher [37] submitted 40 cats to molecular analyses and detected *C. burnetii* DNA in the birth fluid of 3 (7.5%) animals.

Serological investigations have been carried out in Europe as well. A 61.5% seroprevalence was detected in the United Kingdom when a survey evaluated the circulation of *C. burnetii* among foxes, rodents, and cats; rodents were supposed as an important source of infection for cats, as well as for foxes, but the lower prevalence found in rodents (17.3%) suggested that felines can acquire coxiellae also from other sources [38].

Candela and collaborators [39] investigated the exposure of free-living European wildcats (*Felis sylvestris sylvestris*) to C. burnetii in central Spain; they found a 33.3% seroprevalence and the results, although related to a very small number of tested animals (three positive/nine tested), suggested that wildcats should be considered a part of the epidemiological cycle of C. burnetii, in the same way as stray and pet cats [39].

In the period 2005–2007, sera from 291 free-roaming cats living in southern Spain were analyzed, and 108 (37%) had antibodies to *C. burnetii* phase I and II; adult cats were more likely to be seropositive than young individuals, and seropositivity was correlated with urban areas, human population size, and peri-urban areas with shrubs, but not correlated with agricultural landscapes [40].

An observational descriptive study was conducted in Portugal at two time points nine years apart, 2012 (29 cats) and 2021 (47 cats). Sera obtained from dogs and cats (total 294 sera) were tested for *C. burnetii* antibodies using a commercial ELISA adapted for multi-species detection; whereas a 17.2% prevalence was found among cats in 2012, when a higher percentage of animals from rural areas were analyzed, no positive cats were detected in 2021 [41]. Moreover, a molecular survey on reproductive tissues and/or endometrial swabs from 107 cats detected no positive cats [41].

Conversely, a molecular study carried out in Italy found *C. burnetii* DNA in blood samples of 29.4% (25/85) stray cats [42].

Exposure to *C. burnetii* is also possible in cats living in limited environments such as catteries; Shapiro et al. [43], in Australia, detected different seroprevalences in relation to investigated animals: 9.3% (35/376) in cattery-confined breeding cats, 1% (2/198) in pets, whereas no feral (0/50) and shelter cats (0/88) had specific antibodies [43]. These findings induced to suppose that in a given confined environment, cats have a common source of infections, and maybe the pathogen can be transmitted from a cat to another one.

An overall 13.1% (19/145) seroprevalence was found in cats from New South Wales (Australia), with values that varied significantly between communities and the highest prevalence in communities within 150 km of a 2015 human Q fever outbreak [44]. However, when the same blood samples were submitted for molecular analyses, no *C. burnetii*--positive cats were detected [44].

Prevalence values obtained in the serological surveys carried out to evaluate the circulation of *C. burnetii* in feline populations worldwide are summarized in Table 1.

## 4. Sources of Infection

### 4.1. Arthropods

*Coxiella burnetii* can be considered an arthropod-borne pathogen, in view of its detection in over 40 species of hard ticks and 14 soft tick species collected from vegetation and domestic and wild animals. Ticks acquired *C. burnetii* through a blood meal from an infected animal at all stages of their development or transovarially [13].

*Coxiella burnetii* has been detected in several tick tissues, including midgut, hemolymph, Malpighian tubules, salivary glands, and ovaries [45]. Ticks can excrete high number of coxiellae in their feces (up to 10^10^ organisms per gram of feces) [46]; this finding emphasizes the potential risk of tick-borne infection posed by tick excreta, through inhalation (e.g., among shearers), direct contact (e.g., using bare hands to crush a tick), or tick bites [13,47].

Ticks play a meaningful role in the natural cycle of transmission of *C. burnetii* among wildlife and between wild animals and livestock [48]. Cats harbor ticks more rarely than dogs, because they usually live in environments where ticks are less present, do not frequent wild areas with their owners, and, furthermore, remove ticks during self-grooming. Therefore, tick bites are not a relevant route of *C. burnetii* transmission for cats. Nevertheless, inhalation of tick excreta can be source of infection also for these domestic animals [13].

The cat flea *Ctenocephalides felis* is a feline hematophagous arthropod, extremely common in many temperate and tropical regions; it also infests dogs, opossums, raccoons, and rats and represents a great majority of fleas in human homes [49]. The cat flea is known to be vector of different pathogens, mainly *Bartonella henselae* and *Rickettsia felis* [50]. The role of fleas in *C. burnetii* transmission is not clear; some authors do not consider cat fleas as vectors of *C. burnetii* between companion animals and humans, because the bacteria were not detected in analyzed arthropods [51,52]. Recently, the role of fleas has been reconsidered: 1.21% of fleas *C. felis* resulted PCR positive for *C. burnetii* in China [53], but also, previous investigations detected this pathogen in *C. felis*. For example, *C. burnetii* has been detected in 2 (1 *Xenopsylla cheopis* and 1 *C. felis*) of the 987 examined fleas in a study conducted in Egypt [54]; furthermore, Psaroulaki et al. [55] detected *C. burnetii* DNA in 2/3 pools of fleas *C. felis* removed from foxes and 6/41 pools of fleas collected from rats. *Ctenocephalides felis* was not proven to be a competent host. However, the ability of fleas to infest different animal species increases the possibility to acquire *C. burnetii*; the detection of the pathogen in fleas removed from rats enhances the opinion that rats act as potential reservoirs of *C. burnetii* [56] and suggests that they represent a source of infections for cats, not only when they are caught and ingested.

### 4.2. Pet Food

The presence of *C. burnetii* DNA in raw meat packages for pet consumption has been demonstrated. Shapiro et al. [57], in a study carried out in Australia, found the pathogen in kangaroo meat packages, while analyzed samples labelled as non-kangaroo meat did not harbor coxiellae; in addition, they detected *C. burnetii* DNA in samples primarily composed of offal (heart, liver, kidney, stomach, intestines, and bone) more frequently than in skeletal muscle meat samples.

The involvement of kangaroo meat as a potential source of the Q fever agent has also been suggested by Ma and colleagues [44] on the basis of data obtained during a serological and molecular investigation on cats and questionnaires proposed to the owners. Among 145 owned cats tested by indirect immunofluorescence assay, 19 (13.1%) had antibodies against *C. burnetii*, although no specimens (whole blood, reproductive tissue, or swab) from the positive animals were PCR positive for the same pathogen. Seroprevalence varied significantly between communities and was highest in communities within 150 km of a 2015 human Q fever outbreak; however, feeding raw kangaroo was identified as a relevant risk factor for exposure to coxiellae. In fact, it was associated with an increased likelihood of seropositivity in cats, with 5 of 16 cats (31.3%) fed raw kangaroo testing seropositive compared to 0 of 54 cats not fed kangaroo [44]. Most kangaroo fed to pets was sourced by pet owners themselves from hunting, roadkill, or other sources rather than store bought. Circulation of *C. burnetii* in kangaroo populations in Australia has been demonstrated [58,59]. This suggests the risk of infection not only for cats, but also for people during manipulation procedures of carcasses and meat through aerosolizing of bacteria.

### 4.3. Milk

Ingestion of raw milk from infected ruminants is considered a common oral route of infection for cats [30]. However, this route primarily applies to cats living on farms where cattle, sheep, and goats infected by *C. burnetii* are present, and the owners give contaminated raw milk to their pets. On the other hand, milk given to household cats living far from farms is usually sourced from the market, where pasteurized and UHT (ultra-high-temperature) milk is commercialized [60].

### 4.4. Preys

Rodents are considered natural reservoirs of this pathogen [61], so it is plausible that they represent a significant source of infection for cats. Rodents are frequently found in countryside areas as well as urban areas, especially where food waste is accumulated. Cats may prey on rodents, and in doing so, they can become infected. Additionally, cats can also get infected by coming into contact with feces, urine, and secretions from rodents. A study carried out in stray cats from northern Italy detected 29.4% of *C. burnetii* bacteremic animals, whereas ticks removed from the same cats were PCR negative for the pathogen. Considering that all cats lived in urban areas far from livestock, it was supposed that rodents were the main source of coxiellae [42].

Similarly, it is supposable that cats can acquire coxiellae when hunting infected birds. Domestic and wild birds, including migratory species, have been found to harbor *C. burnetii* in several investigations. Birds have been attributed a role as vectors of coxiellae and other pathogens to mammals, primarily due to environmental contamination by avian feces [62,63]. Pigeons and passeriformes, in particular, are commonly found in urban areas and contribute to contamination through their droppings. These birds have been found to carry *C. burnetii* on multiple occasions and have been linked to Q fever outbreaks in humans [64,65,66,67].

Additionally, common lizards are normal prey for cats. While reptiles are considered susceptible to *C. burnetii* infection [68], there are currently no available data on the detection of this pathogen in lizards in the literature. However, it cannot be ruled out that lizards living in areas where Q fever is present may acquire coxiellae from the environment, especially considering that these bacteria can persist in the soil for a long time.

## 5. Pathogenesis and Clinical Forms

It is supposable that the pathogenesis of Q fever in cats is similar to that established for other mammals.

*Coxiella burnetii* enters through oral and inhalatory routes and spreads to different organs, with a predilection for the genital apparatus. In particular, *C. burnetii* has a predilection for entry, replication, and persistence in macrophages, but in the host, injured tissues include vascular endothelium, respiratory, renal tubular, and serosal epithelia [3].

When the pathogen enters through inhalation, animals usually develop pulmonary inflammation with respiratory signs. Infection after ingestion of contaminated material requires a higher amount of coxiellae. It has been supposed that cats may act as passive excretors of coxiellae, without developing infection, after entry of bacteria through the oral route; in fact, cats that have drunk infected milk were found to be serologically negative for *C. burnetii*, although the pathogen was detected in their feces [30].

The first specific data about Q fever in cats come from experimental infections carried out by Gillepsie and Baker in 1952 [69]. The researchers infected cats by subcutaneous inoculation, feeding them infected yolk sacs, and exposing them to naturally infected cats; no animals showed clinical signs, even though they developed specific antibodies, and *C. burnetii* was found in blood for up to one month and in urine for two months [69,70].

*Coxiella burnetii* has an affinity for female reproductive apparatus, in particular, the uterus, in all mammals, and it colonizes the placenta in large numbers. The pathogen has been detected in different genital tract tissues of pregnant and not pregnant cats. Nagaoka et al. [71] cultured *C. burnetii* from deep in the vagina close to the uterine cervix of nine (9/29, 31.8%) cats, two without clinical signs, two with fever and abortion, one with respiratory disorders, one with fever, one with a mammary tumor, one with cutaneous injury, and one with peritonitis. Cairns et al. [27] detected *C. burnetii* DNA in uterine tissues, but not in vaginal samples of cats. These findings lead them to suppose that coxiellae have site-specific preferences for their residence and replication, but also that organism numbers can vary in different genital tract tissues, with the amount of *C. burnetii* DNA in the vagina not detectable by PCR. The difference in bacteria numbers could be dependent on the state of the estrous cycle, as also suggested for sheep [72].

Moreover, delivery is the event during which the highest number of bacteria is excreted, similar to what is observed in ruminant species. Asymptomatic parturient queens shed a huge number (10^9^ per g of tissue) of bacteria into the environment during parturition [3]. Abdel-Moein et al. [37], in Egypt, examined for *C. burnetii* by PCR in pregnant, parturient, and post-parturient pets; 3 out of 40 (7.5%) analyzed cats were positive; in particular, all pregnant and post-parturient cats were negative; and the positive samples were found in the birth fluids of parturient queens (3/19, 15.8%).

Similarly, Kopecny et al. [23] found that uterine and ovarian tissues of 11-weeks-post-parturient cats incriminated in a human Q fever outbreak in Australia were negative; in addition, Ma et al. [44] did not detect *C. burnetii* in a portion of placenta collected from six pregnant queens. On the contrary, *C. burnetii* was found in feline uterus at 3 and 8 weeks post-parturition during outbreaks in Canada [17,18].

It is well known that coxiellae reach the udders of ruminants, sometimes causing mild mastitis, and are excreted in milk [73]. Boden et al. [74] detected *C. burnetii* DNA in the colostrum/breast milk of an infected woman, suggesting that coxiellae, after the bacteremic phase, are localized in the udders of all mammals. Reports about localization of *C. burnetii* and/or mastitis due to this pathogen in nursing cats are not available; however, the presence of *C. burnetii* in feline milk seems possible and could be a further source of infection for kittens.

The main Q fever clinical signs observed in cats and humans are summarized in Table 2.

## 6. Diagnosis

Diagnosis of Q fever can be carried out through indirect and direct methods. In all cases, no protocols specific for diagnosis in cats are available.

Serological diagnosis includes methods with different sensitivity and specificity characters. The World Organization for Animal Health (OIE) reports the complement fixation test (CFT) as the reference test for serological diagnosis of Q fever [76]; however, although CFT is a highly specific test, indirect immunofluorescence assay (IFA) and enzyme-linked immunosorbent assay (ELISA) are more sensitive methods.

CFT is a complex and time-consuming test; it detects both anti-phase I and II antibodies, but sera should be heat inactivated before testing against phase I antigens [77]. It has been observed that a prozone phenomenon may be present in sera from humans with chronic Q fever with consequent false-negative results [78,79].

IFA is considered the gold-standard method because it is the simplest and at the same time, one of the most accurate tests. It is able to distinguish between antibodies to phase I and those to phase II. It is largely employed in serological diagnosis of human Q fever, but it is also suitable for diagnosis in animals, mainly when few individuals have to be investigated. Numerous serological surveys to detect the circulation of *C. burnetii* in feline populations, as well as diagnosis in single animals, have been carried out using IFA [28,29,31,32,34,35,36,44].

ELISA is able to detect both anti-phase I and II antibodies, and is sensitive but laborious [79]. Commercial kits are available for the diagnosis of Q fever in humans and farm animals, but not for cats and dogs; therefore, the test has to be adapted to these investigated animal species [30,33,38,39,40,41,43].

Western immunoblotting has also been reported as a specific and sensitive method for the diagnosis of Q fever in humans [80]. It is based on the entire spectrum of antigens of *C. burnetii* and allows differentiation of the humoral response to a number of different antigenic components of this microorganism [79]. It is expensive, very laborious, and time-consuming, and is thus not suitable for screening in animal populations.

Information about interpretation of the serology of *C. burnetii* infection in cats is limited. Serological tests have not been standardized for cats, and the cut-off values have not been established for these animals. Tissot-Dupont et al. [81] recommend, as IFA cut-off values, titers of anti-phase II IgG of ≥200 and titers of anti-phase II IgM of ≥50 for the diagnosis of human acute Q fever, and titers of anti-phase I IgG ≥ 800 for the diagnosis of human chronic Q fever. Furthermore, in human diagnosis, a CFT titer of 1:40 is considered diagnostic for acute Q fever, while a 1:200 titer of antibody to phase I is diagnostic for chronic Q fever [78].

Usually, lower antibody titers have been considered as cut-offs in cats. An antibody titer of ≥1:16 was taken as positive by Morita et al. [31]; Matthewman et al. [36] considered a 1:40 titer as the cut-off value; Kilic et al. [34] considered cats with a result of ≥1:64 for phase II antibodies as positive. Higgins and Marrie [29], testing cats for antibodies to phase I and phase II *C. burnetii* antigens with IFA, detected positive cats with antibodies ranging from 1:8, considered as the cut-off, to 1:2048 against phase I and 1:1024 against phase II.

An accurate diagnosis, mainly in cats with reproductive disorders, should also include the detection of *C. burnetii* in biological samples. Cultivation of the pathogen requires cell cultures or embryonated hen eggs; all procedures are very expensive and time consuming and must be carried out only in biosafety level 3 laboratories due to the high infectivity of *C. burnetii* [72]. Molecular detection by polymerase chain reaction (PCR) is largely employed for direct diagnosis in all animal species. Vaginal swabs, placentae, aborted fetuses, feces, and urine from cats are the specimens used to investigate. The method is very sensitive, and protocols targeting different genes are available [79].

A fluorescent in situ hybridization (FISH) assay targeting 16S ribosomal RNA and immunohistochemistry have been employed for the detection of *C. burnetii* in ruminants’ tissue samples [82], and they could be similarly used for the diagnosis of Q fever in cats.

## 7. Treatment and Prophylaxis

*Coxiella burnetii* is inhibited by tetracyclines, chloramphenicol, rifampicin, and only to a very limited extent, by penicillin, streptomycin, erythromycin [83]. However, tetracyclines and oral doxycycline (10 mg/kg/die for 2 weeks) are suggested for treating cats [3,84].

Vaccines to prevent Q fever in cats are not available. To minimize the risk of infection in cats, treatment against hematophagous arthropods are pivotal. Furthermore, cats should be kept far from products (raw milk, urines, feces, genital secretions, placentae, aborted fetuses) originated from *C. burnetii*-infected animals.

Control measures against commensal rodents by maintaining good hygiene conditions and using traps are useful to reduce occasions of predation.

Owners should pay close attention to queens with reproductive disorders and should request specific laboratory diagnosis to promptly initiate treatment and preventive measures if an animal is found to be infected by *C. burnetii.* Similarly, breeders and veterinarians must exercise caution, including wearing masks, goggles, and gloves, when attending to cats during abortion or delivery and in the subsequent days. Additionally, measures to prevent and reduce environmental contamination after abortion or parturition are necessary. Placentae, fetuses, and contaminated materials should be appropriately disposed of, while environments and instruments must be thoroughly disinfected [85].

## 8. Conclusions

Q fever is a zoonosis that can cause severe disease in animals and humans. Although farm ruminants and their products are traditionally considered the main sources of infection for people, infected pets can transmit *C. burnetii* to owners, breeders, veterinarians, and other individuals who come into contact with them.

Cats can play a role in the epidemiology of Q fever, although not as frequently as ruminants. They can act as asymptomatic reservoirs, shedding coxiellae during normal delivery. Additionally, cats can be affected by the pathogen and exhibit clinical signs, with abortion being the most significant. In all cases, infected cats can transmit the infection to humans through direct contact, and they also contribute to environmental contamination.

Breeders and veterinarians should always take preventive measures during activities involving queens, whether they are for abortion, caesarean section, or normal delivery. Additionally, veterinarians must inform owners about the risk of infection associated with parturient queens and emphasize the importance of preventive measures, especially for children, pregnant women, the elderly, and individuals with compromised immune systems.

## Figures and Tables

**Table 1:** Prevalences for *Coxiella burnetii* detected in different surveys, in relation to geographic area, feline population, tests, and antigens.

Country	Examined Population	Test	Antigen	Prevalence (n°Positive/n°Examined)	References
California (USA)	Pound cats	Capillary agglutination	phase I	19.8%	[25]
California (USA)	Stray cats	Microagglutination	phase II	9% (7/80)	[26]
Colorado (USA)	Pet cats Shelter cats	PCR	-	8.5% (4/47) 0% (0/50)	[27]
Canada	Domestic cats	IFA IFA	phase I phase II	24.1% (52/216) 6% (13/216)	[28]
Canada	Domestic cats	IFA	phase I and II	6.2% (6/97) 19.2% (20/104)	[29]
Canada	Pet cats Feral cats Farm cats	ELISA	phase I and II	0% (0/73) 0% (0/52) 3.4% (2/59)	[30]
Japan	Domestic cats	IFA	phase II	16% (16/100)	[31]
Japan	Stray cats Pet cats	IFA	nr	41.7% (15/36) 14.2% (44/310)	[32]
Korea	Pet cats	IFA	nr	8.6% (10/116)	[32]
Iran	Stray cats Pet cats	ELISA	phase I and II	22.35% (19/85) 11.53% (9/78)	[33]
Turkey	Stray cats	IFA	phase II	4.9% (7/143)	[34]
Thailand	Domestic cats	IFA	phase I and II	0.51% (2/390)	[35]
South Africa	nr	IFA	phase II	2% (1/52)	[36]
Zimbabwe	nr	IFA	phase II	13% (15/19)	[36]
Egypt	Pet cats	PCR	-	7.5% (3/40)	[37]
United Kingdom	Domestic cats	ELISA	phase I and II	61.5% (16/26)	[38]
Spain	Wildcats	ELISA	phase I and II	33.3% (3/9)	[39]
Spain	Free-roaming cats	ELISA	phase I and II	37% (108/291)	[40]
Portugal	Domestic cats	ELISA	phase I and II	17.2% (5/29) 2012 0% (0/47) 2021	[41]
Portugal	Domestic cats	PCR	-	0% (0/107)	[41]
Italy	Stray cats	PCR	-	29.4% (25/85)	[42]
Australia	Cattery cats Pet cats Feral cats Shelter cats	IFA and ELISA	phase I and II	9.3% (35/376) 1% (2/198) 0% (0/50) 0% (0/98)	[43]
Australia	Domestic cats	IFA	phase I and II	13.1% (19/145)	[44]
Australia	Domestic cats	PCR	-	0% (0/145)	[44]

Legend. IFA: indirect immunofluorescence assay; ELISA: enzyme-linked immunosorbent assay; PCR: polymerase chain reaction; nr: not reported.

**Table 2:** Main Q fever clinical signs observed in cats and humans.

Host	Clinical Signs	References
Cats	Abortion premature delivery stillbirth and perinatal mortality fever anorexia lethargy respiratory disorders splenomegaly	[3,71]
Humans	fever sore throat chills headache lethargy myalgia nausea, vomiting diarrhea, abdominal pains chest pains pneumonia osteomyelitis, osteoarthritis hepatitis cholecystitis endocarditis, myocarditis, pericarditis encephalitis acute lymphadenitis exanthema placentitis, premature delivery spontaneous abortions	[75]

## Data Availability

The data presented in this study are contained within the article.
